# Food Composition at Present: New Challenges

**DOI:** 10.3390/nu11081714

**Published:** 2019-07-25

**Authors:** Maria Kapsokefalou, Mark Roe, Aida Turrini, Helena S. Costa, Emilio Martinez-Victoria, Luisa Marletta, Rachel Berry, Paul Finglas

**Affiliations:** 1 Department of Food Science and Human Nutrition, Agricultural University of Athens, 11855 Athens, Greece; 2 EuroFIR AISBL Executive Board, 1050 Brussels, Belgium; 3 Research Centre for Food and Nutrition (CREA-Food and Nutrition), CREA-Council for Agricultural Research and Economics), 00178 Rome, Italy; 4 Department of Food and Nutrition, National Institute of Health Dr. Ricardo Jorge, I.P., 1649-016 Lisbon, Portugal; 5 REQUIMTE, LAQV/Faculty of Pharmacy, University of Porto, 4050-313 Porto, Portugal; 6 Institute of Nutrition and Food Technology “José Mataix”, University of Granada, 18016 Armilla (Granada), Spain; 7 Quadram Institute Bioscience, Norwich, Norfolk NR4 7UA, UK

**Keywords:** food composition data, food description, food matching, data quality, dietary assessment, research infrastructure

## Abstract

Food composition data is important for stakeholders and users active in the areas of food, nutrition and health. New challenges related to the quality of food composition data reflect the dynamic changes in these areas while the emerging technologies create new opportunities. These challenges and the impact on food composition data for the Mediterranean region were reviewed during the NUTRIMAD 2018 congress of the Spanish Society for Community Nutrition. Data harmonization and standardization, data compilation and use, thesauri, food classification and description, and data exchange are some of the areas that require new approaches. Consistency in documentation, linking of information between datasets, food matching and capturing portion size information suggest the need for new automated tools. Research Infrastructures bring together key data and services. The delivery of sustainable networks and Research Infrastructures in food, nutrition and health will help to increase access to and effective use of food composition data. EuroFIR AISBL coordinates experts and national compilers and contributes to worldwide efforts aiming to produce and maintain high quality data and tools. A Mediterranean Network that shares high quality food composition data is vital for the development of ambitious common research and policy initiatives in support of the Mediterranean Diet.

## 1. Introduction

Food composition data is important for a wide range of stakeholders and users including researchers, public food and health policymakers, healthcare professionals, industry (food, agriculture, software developers), consumers and for educational purposes. Each of these groups has different uses for the data and individual users within the groups may have very different requirements and expectations of the data. For many uses, food composition data is linked to food purchase or consumption data to enable calculation of nutrient intake and assessment of quality of the diet. Traditionally, food composition data has mainly been used for research and to underpin national dietary monitoring programs related to policy.

The major source of food composition data for both non-commercial (research, academic, public operators, educators) and commercial use is authoritative national food composition datasets. These datasets are typically produced and published by national governmental bodies but may also be produced by research institutes and other non-governmental agencies. The data may be published as either reference datasets, which may be incomplete in terms of foods and nutrients included, or user datasets that have been modified from the original reference datasets. Researchers in dietary science (and some industry users) tend to use data at a detailed level and often want to use modified or extended versions of published datasets. Research users often access composition data through datasets that have been embedded in a wide range of software tools and in many cases, users may not be aware of the original data sources or of limitations in the data provided, including whether or not the software includes up-to-date values [1]. 

Nutritional information is also available for many branded food products, particularly those produced by large manufacturers and sold by major retailers. While this data is easily available for individual products (through retailer and manufacturer websites), compiled datasets have not been widely used for research purposes. Datasets for branded products are commercially available and some manufacturers or retailers may provide product datasets, but they are either expensive and/or technically difficult to use. However, there is potentially significant research value in these datasets being more readily available for academic research purposes. The United States Department of Agriculture (USDA) has integrated a branded food database in the national nutritional database [2]. In addition to nutrient data, researchers are increasingly interested in using data on non-nutrient bioactive compounds. Many compounds have been found to have beneficial or toxicological health effects in humans and data on bioactive compounds has been compiled and published in online databases [3,4,5,6]. The research use of this data is rapidly increasing in response to public awareness and the wider availability of published data. 

Nationally representative datasets are produced in most developed countries and increasingly in developing countries, although coverage of foods and nutrients may be more limited. Datasets are typically produced, managed and published by groups with a high level of sustainable expertise in food composition. Sources of data used within the datasets include: analytical data produced specifically for the dataset using standard methods in accredited laboratories, data from scientific or grey literature, calculations from ingredients of composite foods, manufacturers’ data and data from other national datasets. Data is managed and published using a range of data handling tools, most commonly relational databases, although some datasets are still compiled and published in spreadsheet format. Although data standards do exist, the structure of food composition data is not yet fully standardized and depends on the source of the data and the knowledge and expertise of data compilers.

There have always been challenges in harmonizing approaches to acquisition and publication of nutrition, health and lifestyle data, and while data processing and publication in electronic form makes large datasets easier to handle and more accessible, the challenges related to data quality, data exchange formats and documentation have increased. Many international projects and research networks have initiated standardization of methods to collect, manage and publish food composition data, but data standardization has not kept pace with fast moving developments in information and communications technology. The ability to generate and exchange large amounts of data has therefore highlighted existing limitations in data structure. A European Food Safety Authority (EFSA) project to produce a European food composition dataset for use with the EFSA Comprehensive European Food Consumption Database highlighted limitations in combining European food composition data from different countries [7]. 

This paper identifies the main challenges in producing and compiling high quality food composition data and reviews progress on harmonization and standardization of data and developments in tools and software that benefit both compilers and users. Moreover, the paper describes the current state and future challenges of food composition databases (FCDBs) in the Mediterranean countries, particularly Greece, Italy, Spain and Portugal, as discussed during NUTRIMAD 2018, the 12th Congress of the Spanish Society for Community Nutrition that was jointly held with the 4th World Congress of Public Health and Nutrition in Madrid between 24 and 27 October 2018 [8].

## 2. Challenges in Food Composition

Although food composition datasets and other large food and nutrition related datasets (e.g., food consumption data, food trade data) are now more accessible, and technology enables transfer of data between users, methods used to generate and publish data can limit effective use. If data are made available according to the FAIR Data Principles of findability, accessibility, interoperability, and reusability [9], many users could acquire and use data without being aware of limitations and there is a risk that these limitations may influence the way that data analysis is interpreted. In this issue, guidance by the European Open Science Cloud has been delivered and specific research groups have been recognized by the European Commission [10]. There are existing networks of expertise that can help to mitigate these limitations and they can play an important role in providing better quality data that is more likely to be fit for purpose. Food composition data can be derived from a range of sources and most food composition datasets contain values produced in a variety of different ways. Data quality is associated with various factors including: food description, component identification, sample collection, sample handling, analytical method, value documentation and laboratory performance. Systems for evaluating quality of nutrient data have been developed [11,12] but information on data quality is not usually published. Quality of food composition data has been considered as part of a Quality Management Framework for the production and management of food composition data [12].

One of the major problems of food composition datasets has always been providing and maintaining data that reflects the range of foods and nutrient composition of foods that are being consumed. The nutrient content of food changes over time and food composition databases should be continually revised to provide data for new foods and for foods where composition has changed. Nutrient composition may change for reasons including growing conditions, changing agricultural practices, plant breeding developments, changes in processed methods and changes in consumer expectations and preparation [13]. Processed foods are constantly changing as manufacturers try to protect or increase market share and profits and respond to policy changes dictated by a combination of government policies and consumer pressure, e.g., reduction of sugar, salt, saturated and trans fats. In recent years the rate of changes in composition and foods consumed has increased in many countries, in line with increased focus on the role of diet in population health. The need to maintain nutrient datasets is constant but in practice most national datasets are not continually revised and updated versions are published according to resource availability and based on foods that are identified as being the most important contributors to diets. Priorities may be based on food type, e.g., surveys of eggs, meat, fish, fruit or vegetables, or in some cases based on nutrients that are expected to have changed, e.g., sugars, fatty acids, sodium. The recent economic climate has resulted in most compilers having to work with increasingly limited resources, including budgets for analysis, equipment and staff.

The approach to maintenance of national datasets means that a percentage of data will always be old, and some old data will not be representative of foods that are currently consumed. Ideally, data contained within datasets will include information on the date of generation and/or publication and will be validated at the time of publication. Even where information on data validity is available, datasets will almost certainly still contain out of date values and, following publication, validity of the dataset will decrease over time. The impact of out of date values depends on what the data is used for but can be very significant in some cases. One of the main reasons for national food composition tables’ (FCTs) data obsolescence is the lack of funded programs to a) perform analyses and b) to acquire innovative tools for making quicker chemical analysis processes [14]. This issue also causes a non-secondary effect as FCT users try to complete missing data borrowing values from other datasets, so obsolescence can be propagated. Specific strengths, weaknesses and challenges may be identified when considering national or regional FCTs and FCDBs. 

**Figure 1:**
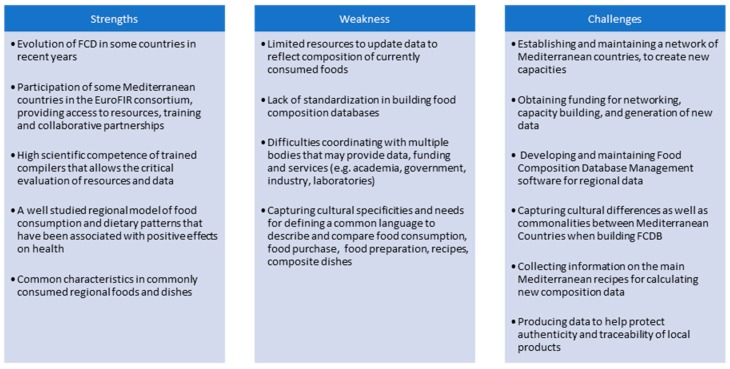
Strengths, weaknesses and challenges of food composition databases (FCDBs) in countries in the Mediterranean region.

The Mediterranean countries are an interesting case study because of the coexistence of European, African and Middle East countries along the Mediterranean region, which provides a good example when considering regional FCTs and FCDBs. Figure 1 is a description of the current strengths, weaknesses and challenges of food composition datasets in the Mediterranean region and was developed during the NUTRIMAD 2018 conference by compilers responsible for national datasets in the region.

Shared and exploitable unique characteristics in the local food system include respect for diversity in the primary production, preservation of traditional foods and a large number of products that have been assigned as Designation of Origin (PDO) or Protected Geographical Indication (PGI) [15]. Synergies among the Mediterranean countries in development, update and maintenance of FCTs and FCDBs is required in order to overcome, at least partially, the limitations in resources and respond to new challenges of the digitally interconnected world. Intellectual support, sharing of data and best practices may be drawn from participation to networks such as European Food Information Resource (EuroFIR) [16] and International Network of Food Data System (INFOODS) [17]. 

Following the recognition of these common characteristics a consortium of six Mediterranean Sea coast countries (Cyprus, Croatia, Spain, Greece, Italy, Morocco) and Portugal succeeded in 2013 to include the Mediterranean Diet in the list of Intangible Cultural Heritage of UNESCO [18]. This initiative emphasizes and shares internationally the uniqueness, values, and collaboration of Mediterranean countries and creates the potential for exploitation of this dietary model to the benefit of local economies and culture.

Published food composition datasets can be either reference or user datasets [12]. Published datasets are usually a sub-set of reference datasets and may not include all foods and nutrients included in the reference dataset. Some published values may be recalculated for publication of derived values, including recipe calculations and aggregations of branded food data. Reference datasets and user datasets often do not include values for all nutrients for all foods so values may be added to fill gaps. These values may be imputed from other values, estimations from other foods or data sources, or may be logical zeros (e.g., cholesterol, vitamin B12 in plant foods). Some nutrient datasets and bioactive databases only include data for raw foods and do not provide data for cooked and processed foods. These datasets are unsuitable for use in intake calculations because they do not include data for many foods as consumed and do not include data for composite dishes (foods prepared from more than one ingredient). For most purposes it is necessary to adapt published data for specific uses. Most uses will require values for all nutrients to avoid the possibility of missing values being assumed to be insignificant and treated as zero. Some nutrients, e.g., fatty acids and individual sugars, are usually only provided for a limited sub-set of foods so they may also need to be added by users. National datasets do not all include the same nutrients and while the major nutrients will be included, nutrients such as individual sugars, individual fatty acids, and some minerals, trace elements and vitamins may not be included. This is not a problem when users are only using data from one country but can be problematic when data from different countries and sources are combined. Many users of the data are not aware of the limitations of published data, despite detailed documentation being provided for most national datasets. Where users are aware of the need to adapt data, there is often a gap in the knowledge and skills and/or the resources needed to adapt the datasets for correct use.

Datasets of branded foods may be updated continually following agreements between producers and wholesalers and retailers, who often require updated composition data as a pre-requisite for distributing or stocking products. This information is often supplied by a third-party organization that receives information from producers and compiles the data into datasets that are then supplied to retailers for online use. An example is Brandbank [19] who compile and distribute data globally, on a commercial basis, to provide online solutions for retailers. Although the values included in datasets are as up-to-date as possible, the format of the data and consistency of food description pose some challenges to users. The data relate to very specific products, based on Global Trade Item Number (GTIN) code [20], and new products are continually added while others are discontinued. Branded food data usually only provides nutrient values that are required or permissible for food labelling. The EU labelling regulations [21] do provide a standard for how the values are calculated and presented but provision of values for some nutrients (e.g., fibre, starch, mono and polyunsaturated fatty acids) is optional so values for those nutrients will not be provided for all foods. Values for minerals and vitamins can only be included on food labels when a significant amount of the nutrient (15% of nutrient reference value for foods or 7.5% for beverages) is present and, as a result, mineral and vitamin contents of foods are rarely available for branded foods. For uses that require values for those nutrients, e.g., calculation of nutrient intakes, these values must be added to the dataset. It is possible for branded foods to be mapped to generic foods so that mineral and vitamin contents can be estimated but that work requires considerable expertise and technical data management skills. For that reason, branded datasets have not been completed (in terms of nutrient coverage) and fully merged with generic national datasets.

In 2013, European compilers produced a food composition dataset for EFSA that aimed to provide an updated food composition database covering approximately 1750 foods and to expand the dataset to include harmonized information on the most common composite recipes of European countries. The dataset was compiled to be compatible with the EFSA Guidance on Standard Sample Description for Food and Feed [22] and included additional descriptors from the EFSA FoodEx2 classification system [23]. To ensure a complete nutrient dataset was provided, where data was not available in a national database, values were borrowed from another country. The project highlighted a number of significant limitations in the dataset that were related to the need to standardize approaches to compiling and using data to ensure that data is as comparable as possible [7].

Bioactive compound databases are compiled in a similar fashion to nutrient databases, employing a systematic search and selection process utilizing appropriate online searching tools, such as Web of Science. Published databases cannot be described as comprehensive, primarily because new literature is constantly being published and analytical methods for many bioactive components are constantly developing and are not standardized. The main sources for polyphenol composition data (USDA [3], eBASIS [4], Phenol-Explorer [5]) are expert-based curated databases rather than being compiled in an automated way. A dataset of anthocyanins in foods consumed in Australia has also been developed [6]. The literature based, curated approach means that these datasets are labor-intensive to produce, maintain and update, which in turn means they require consistent funding and resources and therefore updating may be limited.

The limitations of compiling datasets mean that there may also be limitations for users that depend on the intended use. ‘Static’ datasets that are included in applications may be out of date when they are used and in many cases data users do not routinely update to newer versions of the data. While updating is technically possible, many users do not have the skilled IT resources or sufficient knowledge of the data to make the necessary updates. For some purposes, e.g., research studies over extended time periods, it may not be desirable to use updated data because nutrient intake may then be changed because of changes in the underlying composition dataset rather than just in the foods consumed.

To overcome these limitations and challenges it is essential that datasets are documented as fully as possible, using international standards, to allow the possibility of reliably exchanging information between datasets. While it is increasingly easy to store and transfer large datasets and make them available online, it is usually necessary to do a lot of work with cleaning and standardizing datasets before data can be accurately and usefully combined. This work relies on the knowledge and training of a relatively limited number of compilers who have the required experience.

## 3. EuroFIR

Traditionally there has not been a standard structure for food composition data because datasets have been compiled independently for publication in country specific printed tables in books and scientific journals. Since the introduction of computerized data compilation and publication, there has been a trend towards more standardized data structures and control of data quality through clear documentation of the data. 

There have been many collaborative projects and networks of food composition data compilers that have aimed to improve consistency and harmonization of composition databases, so that values from different datasets are of comparable quality. European projects such as EuroFOODS, Cost Action 99, the IARC European Nutrient Data Bank project [24,25] and the work of INFOODS [17], all made progress towards more standardized production, compilation and management of data. These and other related projects were used as the basis for the European Food Information Resource (EuroFIR) project.

The EU FP6 and FP7 EuroFIR Network of Excellence (NoE) (2005–2010) and EuroFIR NEXUS (2011–2013) projects [26] aimed to standardize and harmonize food composition data in Europe through improved data quality, database searchability and standards. To further standardize the EuroFIR quality approach, new or existing procedures and tools were developed or adopted for data interchange, food description, component identification, value documentation, recipe calculation and quality evaluation of values. Following the NEXUS project, EuroFIR AISBL was formed in 2009 as an international, member-based, non-profit Association and aims to ensure sustained advocacy for food information in Europe and facilitate improved data quality, storage and access, and encourage wider applications and exploitation of food composition data for both research and commercial purposes.

### 3.1. Data Harmonization and Standardization

EuroFIR AISBL is the European regional data coordinator for INFOODS, the worldwide network of food composition experts aiming to improve the quality, availability, reliability and use of food composition data. EuroFIR and INFOODS have both developed standards for production of harmonized nutrient data and national datasets are becoming more harmonized as more national compilers are trained in the use of standards and are increasingly aware of the need to produce data that is comparable with other datasets. However, accessing and combining data from different datasets is not an easy task, even though the data for individual datasets is easily accessible. EuroFIR produced a range of tools to help data compilers harmonize and standardize data, including procedures for documenting data values, and supported the development and publication of a European standard for food data [27] that was based on the EuroFIR technical standard [28,29]. The European Committee for Standardization (CEN) standard also took into account recommendations of the GS1 Global Data Synchronization Network (GDSN) Trade Item standard Food & Beverages extension [30] used in the retail industry, and the CEN standard is therefore a flexible standard that can support not only food nutrients and bioactives data exchange, but also data on feed and data concerning other food properties (e.g., allergen or micro-organism contents, pH, vitamin retention factors). 

These guidelines, standards and training activities have helped data compilers to improve harmonization and consistency of food composition data but the development of electronic datasets that include data from multiple sources has highlighted the fact, that in many cases, datasets still have significant differences and data comparisons are not straight forward. Differences can occur not only between different datasets but also within datasets because data may be compiled from different sources using different methods. 

### 3.2. Tools for Food Composition Compilation and Use

#### 3.2.1. Thesauri

The CEN standard allows use of a range of different thesauri, but current international training programs are focused on thesauri published by EuroFIR [16] and by INFOODS (especially outside of Europe). While not completely identical, these thesauri have many features in common and are continuing to be harmonized so that European and International standards are compatible. A feature of these, and other thesauri (e.g., EFSA Standard Sample Description for Food and Feed [22], FoodEx2 [23]), is that they can easily be mapped from one to another so that datasets using different thesauri can be exchanged relatively easily.

Available thesauri and classification systems that are commonly used include:

- Food classification and description
- Component (nutrient) identification
- Value documentation
- Value type
- Units
- Matrix unit
- Method indicator
- Acquisition type
- Reference type
- Recipe calculation

Thesauri used for value documentation are relatively standardized and contain commonly used terms that allow consistent and clear expression of nutrient values. Standard thesauri are routinely used by almost all national food composition data compilers because most compilers have been trained through either EuroFIR and/or INFOODS training programs. Data produced outside of national programs, including data published in some scientific journals, is typically less standardized because producers are often unaware of standards that are available. 

#### 3.2.2. Food Classification and Description

Accurate and consistent description of the foods included in datasets is a major determinant of data quality and whether values can easily be used by a range of users with different needs [1,31]. Food aggregation creates a crucial structure for food data processing and the interpretation of dietary assessment results [32]. Compositional data can be provided for raw foods, processed foods and for foods as purchased or as prepared for consumption. The different states of the included foods mean that the description of each food, particularly where differences impact on nutrient composition (e.g., vegetables, raw or cooked; vegetables boiled with or without added salt, fish canned in brine or oil) is very important. Publication of data in book or table form allows for additional descriptive text to be added, distinguishing between foods where necessary, but searchable electronic datasets mean that even where the information is available it may not always be presented alongside the food name. Facetted systems have been developed to describe foods and to distinguish between different foods that may not be fully described by the food name alone, e.g., to describe the plant or animal source of a food in more detail; specify parts analyzed and/or inedible waste; describe food processing such as cooking method, preservation method or addition of ingredients. These systems also aid the comparison of data between different datasets, particularly those from different countries or those that were intended for a different purpose.

A standard food group classification structure is an essential starting point that allows aggregation of foods with similar characteristics (e.g., botanical, trade, consumption) and can enable datasets to be more easily exchanged and compared. It is not essential for the same taxonomies to be used (although it does make data handling easier) and indeed selection of an appropriate taxonomy, usually hierarchical, may depend on the application. Classifications for food identification and description are used in food composition and food consumption data, and various taxonomies are available for use with data related to health (e.g., Medical Subject Headings (MeSH)), and lifestyle (e.g., Compendium of Physical Activities [33]).

The LanguaL (Langua alimentaria) system [34] was developed by the United States Food and Drug Administration in the 1970s and has been modified and adopted for use in European countries as part of the EuroFIR initiative to better standardize approaches for food description used for foods in national FCDBs in European countries [35]. EFSA has also developed a similar system of facet descriptors for use with the FoodEx2 food list [23] that is used as the basis for dietary intake, exposure and risk assessments. LanguaL codes are included in EFSA’s FoodEx2 browser tool to allow easy matching to data that already includes LanguaL codes. The use of such facetted descriptive terms is a powerful tool to aid electronic searching of foods, e.g., for matching composition data to food intake data.

#### 3.2.3. Data Exchange

EuroFIR developed a range of generic tools that are available to users to allow retrieval of food composition datasets and transfer of data between users. The EuroFIR AISBL membership structure provides access to the network data, tools and expertise and is available to users on an individual or organizational basis. The key user tools available through EuroFIR AISBL are:

• FoodEXplorer

**Figure 2:**
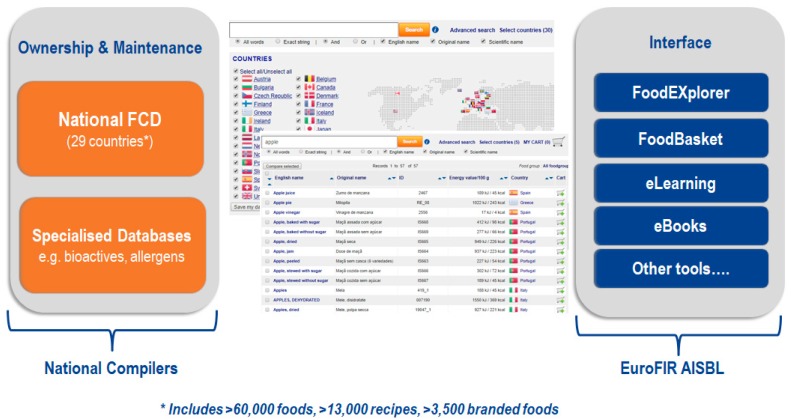
EuroFIR Food Information Platform.

The EuroFIR FoodEXplorer tool is an innovative interface, which can be accessed online and allows users to simultaneously search standardized and specialized food composition databases from across Europe and worldwide (Figure 2). 

FoodExplorer contains data from 29 countries, mainly within Europe but also including Canada, Japan, New Zealand and the USA. The values available within FoodEXplorer are provided by national compilers but are presented in a standardized format and search results can be downloaded into Excel or a bespoke xml format (Food Data Transport Package) for further use. Even though the presentation of results is standardized, there are still some differences in how values are compiled at national level and work to further standardize values and improve usability is ongoing. 

• eBASIS/ePlantLibra

The eBASIS and ePlantLibra databases are specialized datasets of bioactive components in foods and plant food supplements covering their composition, biological effects (mainly human study data), and toxic/adverse effects. Values are compiled from scientific literature and are evaluated by experts.

• FoodCASE

Food composition database management systems (FCDMS) are an essential tool for all data compilers. While data can be compiled using simple spreadsheet approaches, e.g., the INFOODS compiler tool, it is preferable to use a relational database approach that includes full documentation of values and includes both reference and published datasets. Most European compilers have existing FCDMS but many are outdated and can be difficult and expensive to update in line with current operating systems and software. Food Content and System Environment (FoodCASE) is a food composition database management tool, developed in partnership with ETH Zurich and PremoTec (Zurich), that is fully compatible with the EuroFIR technical annex and thesauri, as well as other classification systems and thesauri. Functionality also includes the possibility to publish data directly to a web interface, e.g., the Swiss food composition database [36]. FoodCASE is being continually developed to meet the needs of food composition data compilers worldwide and is a low-cost option available to use subject to a licensing agreement.

#### 3.2.4. Linking to Food Intake Data

Linking food composition data to food intake data is essential for use in dietary monitoring tools. The problem of linking foods as they are described in composition datasets to foods as they are reported in dietary assessment tools can be challenging and is usually a manual and time-consuming task that relies on the judgement of an individual. Automated approaches to food matching have been investigated as part of recent research projects and have been further developed based on combinations of food descriptions and classification codes [37]. The evaluation of this approach concluded that the most relevant requirement for efficient food matching is high quality of food consumption and composition databases. The quality of food information refers to the documentation of the data, including food description, which is the important determinant for food matching, and also component identification and descriptions of data source, sampling, analytical methods, and laboratory performance. The main challenge in food matching is the diversity of data sets to be matched. They differ in the number of food items, the classification and coding system, the level of details provided by descriptors, etc. A food matching algorithm needs to overcome these problems, considering all the information that is available. In the approaches studied, it was possible to combine food information from different food composition and consumption datasets that can easily extend to other types of data (e.g., food purchase data). An approach for linking GS1 data for branded foods to generic food composition data that automatically recognizes food, nutrient and quantity concepts from unstructured text has also been developed [38,39] and tested. 

Estimation of portion size is also challenging and a major source of error in intake estimations. Recent approaches to automate portion size estimation have focused on two types of tools: weighing scales and food portion estimation from images. One approach is to enable Bluetooth communication between small kitchen weighing scales and a mobile app that can capture information on the types and amounts of foods consumed. This approach has been tested and shown to be a feasible and efficient approach [40]. Image recognition techniques to identify foods have also been tested and shown some promise, particularly with relatively simple and distinct foods, although there are still limitations with mixed dishes and more complex foods [41]. A recent study tested the combination of the Fake Food Buffet method with a food matching approach to automate data collection and analysis. The methodology combined fake food image recognition by using Deep Learning and food matching and standardization based on natural language processing. Food matching firstly describes each of the recognized food items in the image and then matches the food items with their compositional data considering both their food names and descriptors [42].

## 4. Collaborative Networks

Collaborative networks of food composition data compilers and users have proved to be an effective approach in dealing with the challenges of producing high quality data and creating and maintaining tools to produce and manage data. Most of these European initiatives have been enabled by EU funded projects with other sources of funding allowing developments to be extended to other areas, e.g., World Health Organization, UK Global Challenges Research Funding program [43]. In addition to funding that enables data to be produced, developed and improved, it is crucial that networks are maintained to enable co-operation, training and exchange of ideas between compilers and data users from different countries and regions of the world. INFOODS and EuroFIR have been providing these opportunities and these networks continue to promote and advocate for the importance of high-quality food composition data that is relevant to specific countries and populations of consumers. EuroFIR AISBL is sustained as a not-for profit members organization that relies on a combination of funding from membership, participation in research projects and commercial activities. INFOODS is funded through the Food and Agriculture Organization of the United Nations. 

Current challenges can be addressed by the formation of new networks that create synergies enabling the development and/or the continuous update of FCTs and FCDBs. For example, a Mediterranean Network expanded to include EU and non-EU countries in the Mediterranean Region would enable the linking and/or exchange of data, resources, infrastructures and best practices. The collaboration of such an initiative with EuroFIR AISBL and other networks will amplify the expected benefits thus supporting its future success and sustainability. A Mediterranean Network on FCTs and FCDBs is vital for the development of ambitious common research and policy initiatives in support of the Mediterranean Diet.

### 4.1. Research Infrastructures

Research infrastructures (RI) are intended to bring together key data and services for the benefit of users. Food composition data has a wide range of users and uses and provision of food composition data that can be linked to tools and services was investigated in the EU-funded RICHFIELDS (Research Infrastructure on Consumer Health and Food Intake for E-science with Linked Data Sharing) project [44]. The overall goal of RICHFIELDS was to bring together the agri-food and nutrition-health domains to collect, collate and connect relevant data, tools and resources to create a single, multidisciplinary Research Infrastructure data platform. Current and emerging RIs and networks were evaluated to assess the feasibility for inclusion in a data platform. The outcomes of the EuroDISH project, which identified RIs and related entities (e.g., platforms, networks and tools) relating to Determinants, Intake, Status and Health (DISH) research areas [45], were used to guide the selection of case-studies. Food composition and food consumption data were included in case studies along with clinical data and use of data in software and apps aimed at improving consumer health. The case studies investigated: data structure; data storage and availability; maintenance and access to data and ethical issues related to provision of and use of data. The outcomes of these case studies informed recommendations for the design for an overarching data platform for a food, nutrition and health RI.

The food composition case-study highlighted the need for data to be harmonized and standardized for efficient data exchange and linking to tools and services. EuroFIR originally envisaged a system where data was held locally by the data producers or publishers and was accessed directly using web services to retrieve the data. In this data transfer model, data can be directly exchanged between computer systems and there is no need for manual transformation of the data. However, the proposed system relied on data providers having access to advanced IT infrastructures with sustainable IT expertise and in practice most producers of national food composition datasets could not provide the necessary resources and further work that was needed. Even though food composition datasets are increasingly harmonized, some reformatting is usually needed for further use. EuroFIR has developed an approach that relies on accessing data that is produced and owned by member organizations, with a centralized server that holds modified versions of the original data. More work is needed to improve data standardization and systems are continually evolving to improve data quality and ease of use. This is particularly important where food composition data is linked to other types of nutritional data such as food intake, contaminants, or to biomarkers related to health. Continued provision of data and services has relied on a committed network of members and users and this will need to continue for effective links to RIs. A sustainable IT and governance infrastructure and access to relevant expertise, including technical, administrative and management, is also essential.

**Figure 3:**
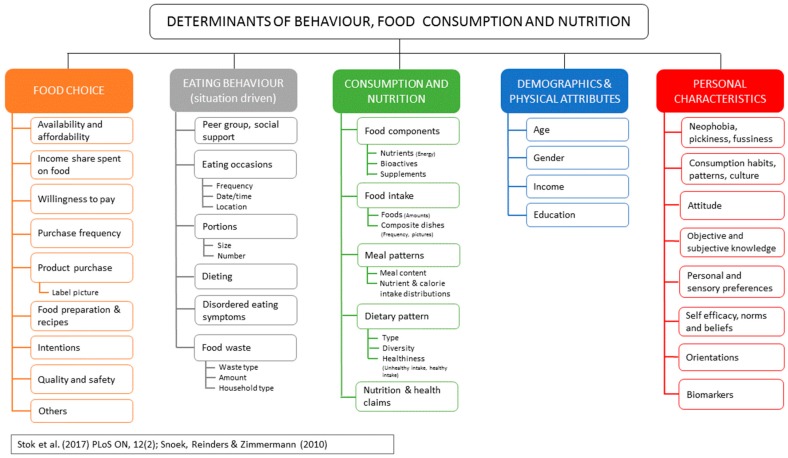
Overview of the determinants of dietary behavior, food consumption and nutrition, including food components. Adapted from Stok et al. (2017) [47] undertaken as part of the Determinants of Diet and Physical Activity (DEDIPAC) Knowledge Hub.

Access to data depends on agreements with data providers and transfer of data and access depends on policies of the data producers and may change over time. A European Innovation Technology program funded project is developing the Quisper (Quality Information Services and Dietary Advice for Personalized Nutrition in Europe) platform [46] that also includes food composition data. The results of these projects will be relevant for all RIs and networks that include food composition data either directly or embedded in tools. An example of how food composition data can fit into a food and health related RI is given in Figure 3. 

The delivery of sustainable networks and RIs, e.g., the proposed Food Nutrition and Health RI [48], will help to increase access to and effective use of food composition data and will help to support continued provision of high-quality data.

### 4.2. Sustainability

Sustainability of food composition data activities has always been a challenge and in many countries maintaining the current level is not guaranteed. Funding from government departments and research funding are vital to sustainability and there is a continual need for advocacy. Despite the improvements in tools that enable effective compilation, a common factor to all food composition databases is that they require both financial resource and human expertise to be maintained and updated and they are therefore vulnerable to loss of staff expertise and organizational restructuring that can directly impact their operations. In the current financial climate, there is a tendency for even well-established compilers to work with reduced funding, meaning that datasets may not be maintained optimally [49]. 

EuroFIR has successfully operated and developed since the formation of EuroFIR AISBL in 2009. It links to other research networks and infrastructures through research and/or commercial projects and the expertise within EuroFIR AISBL allows it to not only provide food composition data but to provide expertise on the use and management of the data for a wide range of users and applications. The EuroFIR AISBL business model has been developed to provide expertise for both producers and users of food composition data, mainly in the research and industry (including food producers and software developers) sectors. The initial EuroFIR AISBL offering has been developed through a range of funded project activities that have led either to an increase in the quantity and/or quality of data provided or to new or improved uses of data. These projects all provided funding to enhance existing data and to improve the quantity, quality and accessibility of data and tools that can be used by data users and providers. Sustainability is achieved through a mixed model of membership fees and ‘on demand’ fees for some licensed data, and consultancies (including participation in research projects). Fees are structured so that larger organizations and businesses pay more compared to SMEs or individual users [16]. 

A key benefit of the approach is that the projects all promote collaborations between project partners, many of them EuroFIR AISBL members, which helped to develop a network of shared expertise and trust that enhances sustainability of the network, data and tools. In recent years, EuroFIR AISBL has been involved in collaborative projects with WHO that have included data compilers from Africa and Asia and this work has increased the network and broadened the links between national compilers. Networks such as EuroFIR AISBL and INFOODS are vital to allow continued cooperation and sharing of data and ideas between individual compilers and organizations. Opportunities to benefit from training and exchange visits are also important to improve data quality and enhance opportunities.

## 5. Conclusions

Food compilation datasets are a vital resource for a wide range of stakeholders. Maintaining up-to-date datasets that are representative of foods currently being consumed is subject to challenges that include: funding to ensure continued development and sustainability; development of tools and software to assist data compilation; development of networks to disseminate data and promote training, development of data structures and infrastructure to enable linking to other networks, platforms and research infrastructures.

High quality food composition data is widely available within Europe, but up-to-date, representative and documented data are lacking in many areas, particularly in developing countries. Food description and classification are vital elements of high-quality data and, while LanguaL and FoodEX2 are well developed systems, the process of documentation is manual and time consuming. There is a need for continued training and for development of automated tools that can help produce more consistent documentation and better enable linking between food composition datasets and other types of related information. Tools for food matching and capturing portion size information have shown promise in linking food composition data and intake data but further development is required. Initiatives to develop research infrastructures in the food and health area are an important development in linking smaller networks, platforms and resources and could provide significant benefits to stakeholders.
